# Supplementary material for: A Selective Histone Deacetylase Inhibitor Induces Autophagy and Cell Death via SCNN1A Downregulation in Glioblastoma Cells
Source: Cancers (Basel). 2022 Sep 19;14(18):4537. doi: 10.3390/cancers14184537 (PMC9496778; doi:10.3390/cancers14184537)

Figure S1. Annexin V staining of LMK235-treated cells

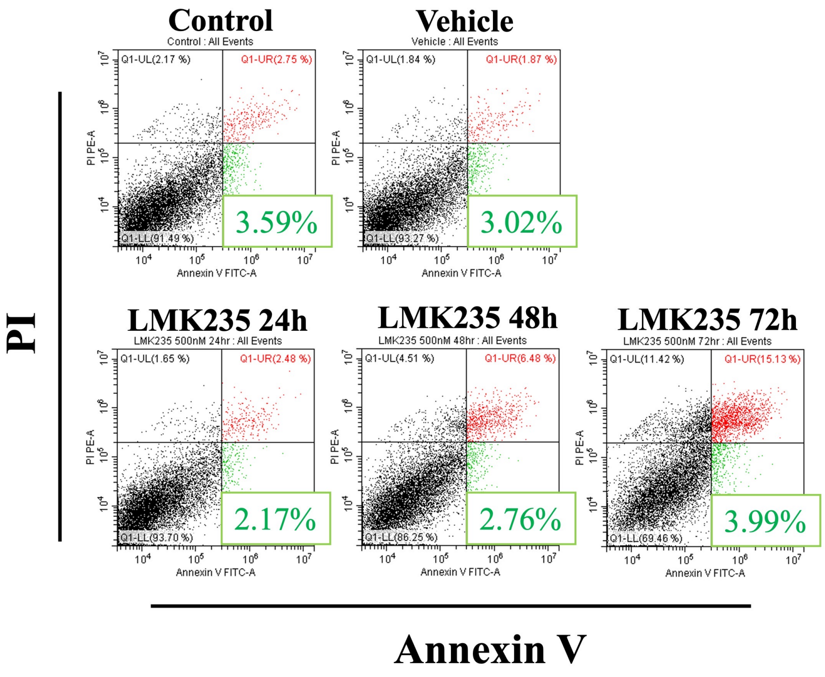

Figure S2. KEGG analysis of LMK235-treated cells

KEGG analysis (LMK235 0.5 μM)

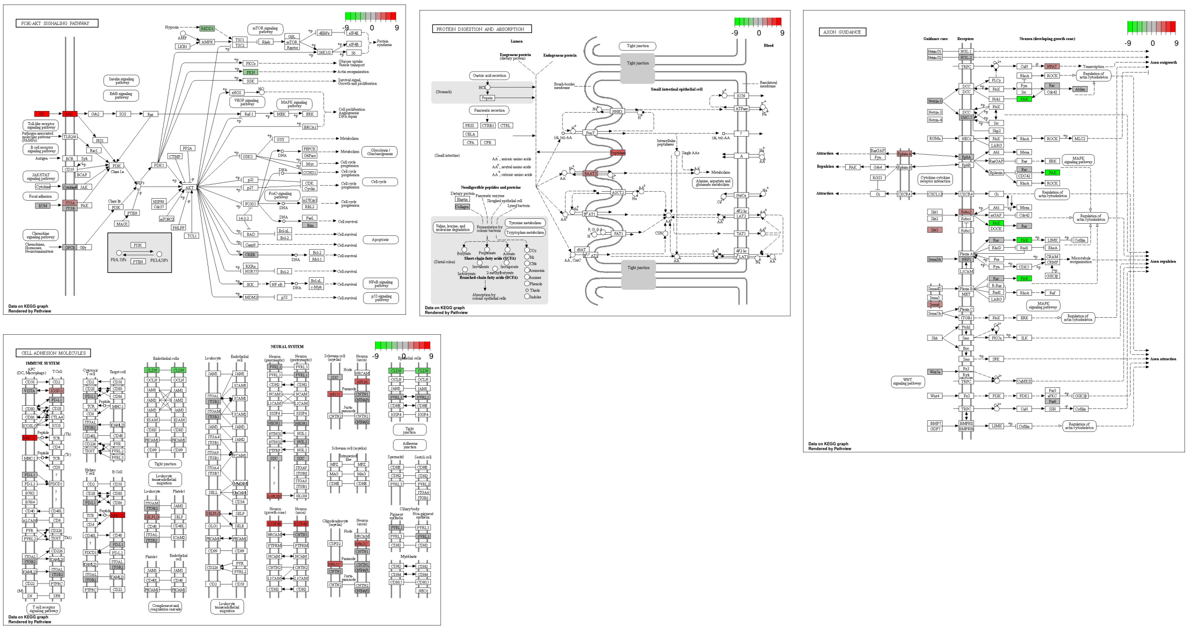

KEGG analysis (LMK235 2 μM)

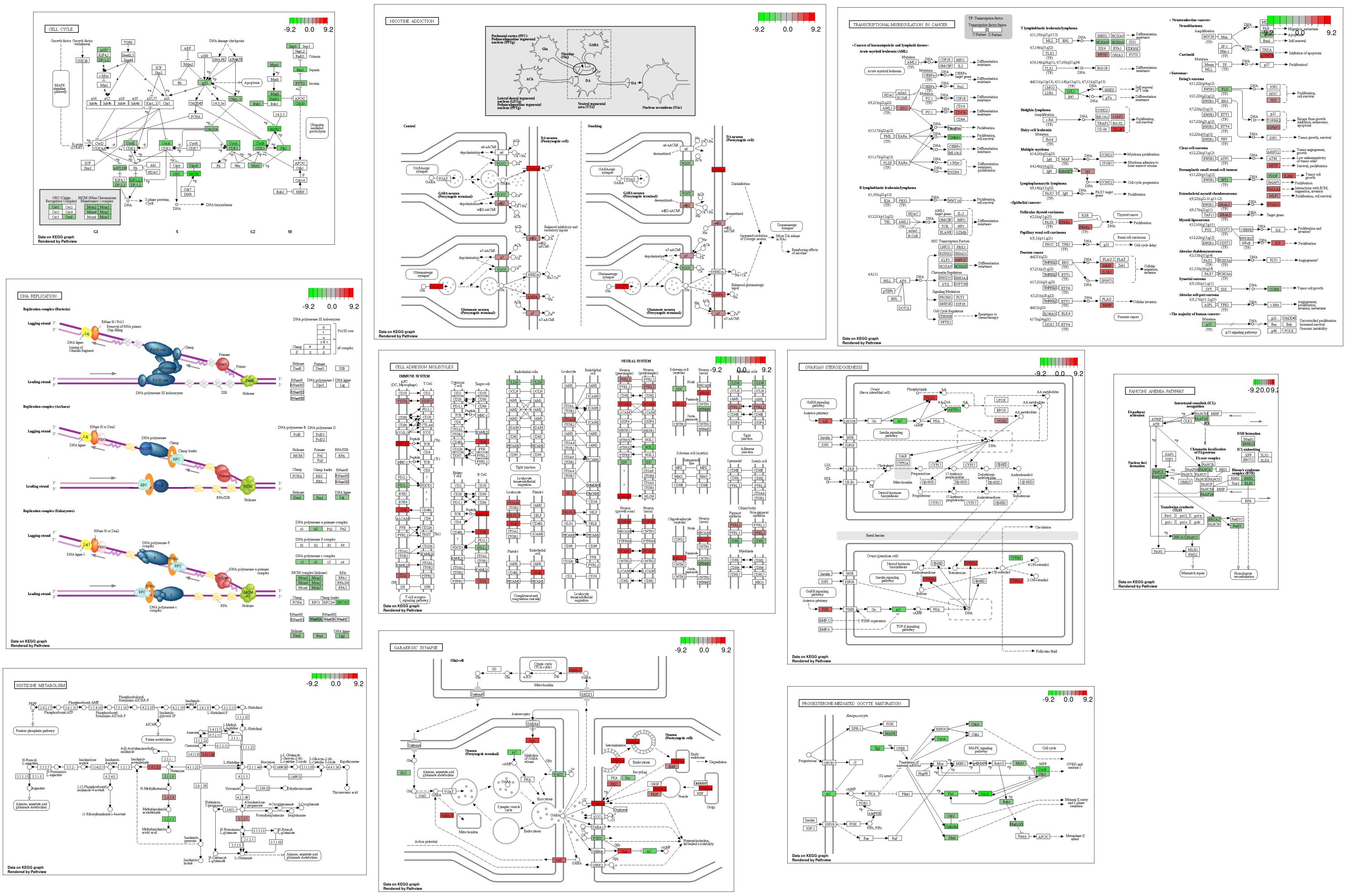

Supplement: Supplementary file 1 [file cancers-14-04537-s001.zip › cancers-1882636-supplementary figures.pdf]
