# Supplementary material for: A Selective Histone Deacetylase Inhibitor Induces Autophagy and Cell Death via SCNN1A Downregulation in Glioblastoma Cells
Source: Cancers (Basel). 2022 Sep 19;14(18):4537. doi: 10.3390/cancers14184537 (PMC9496778; doi:10.3390/cancers14184537)

Whole image of WB in Fig. 2A

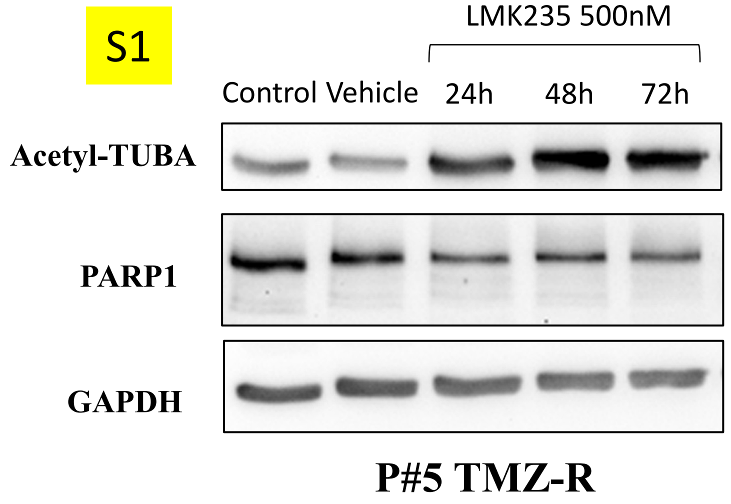

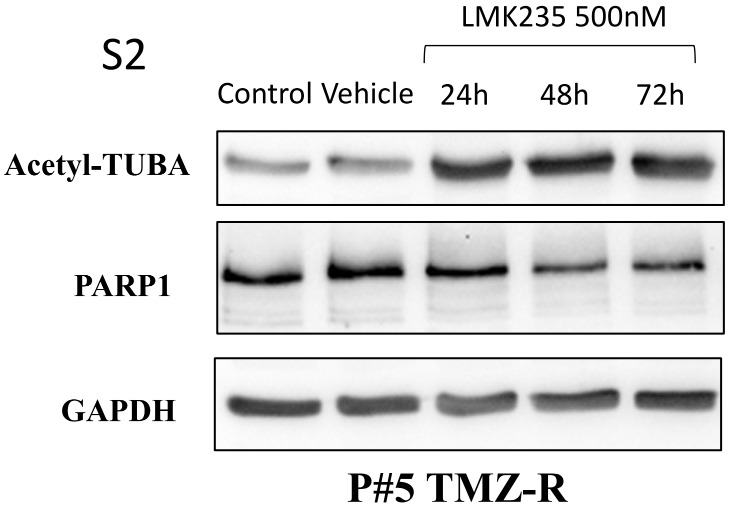

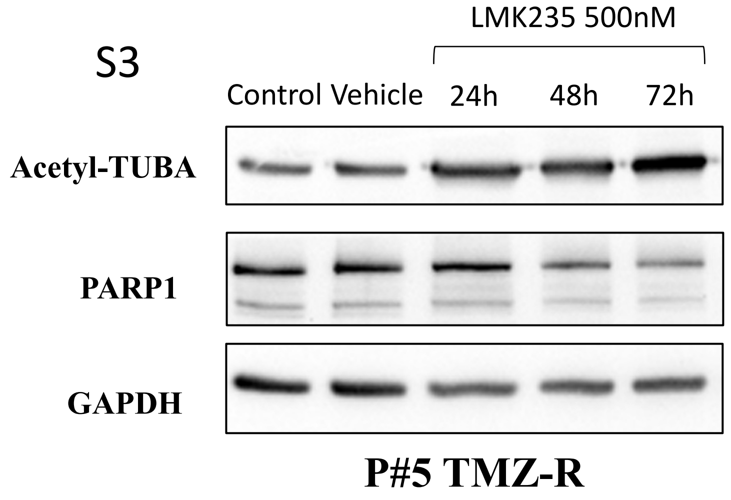

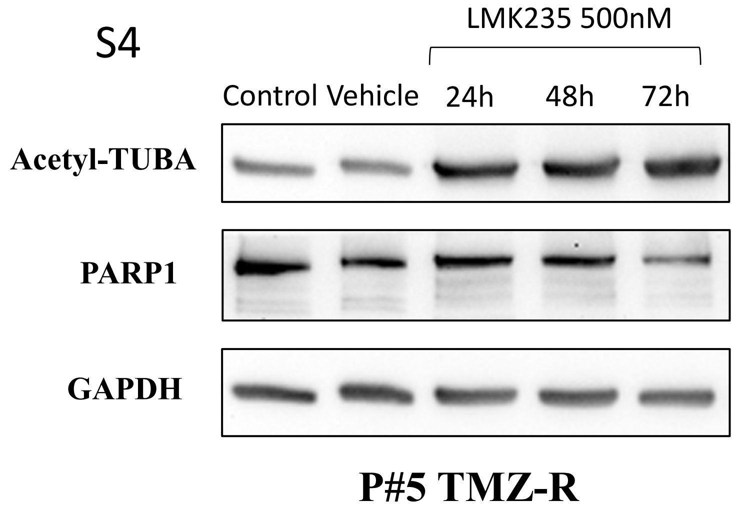

Whole image of WB in Fig. 2D

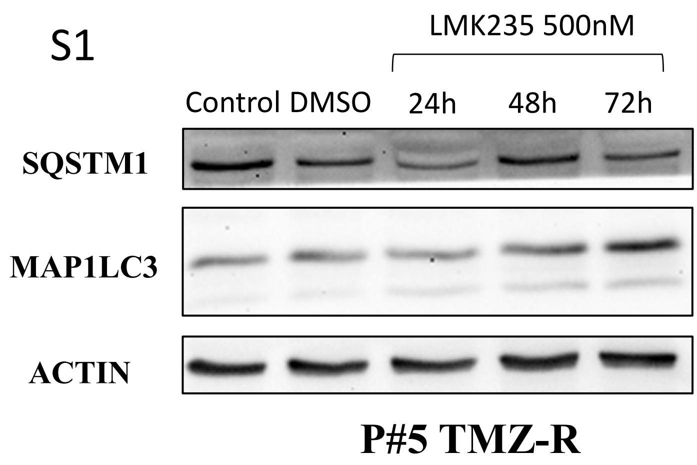

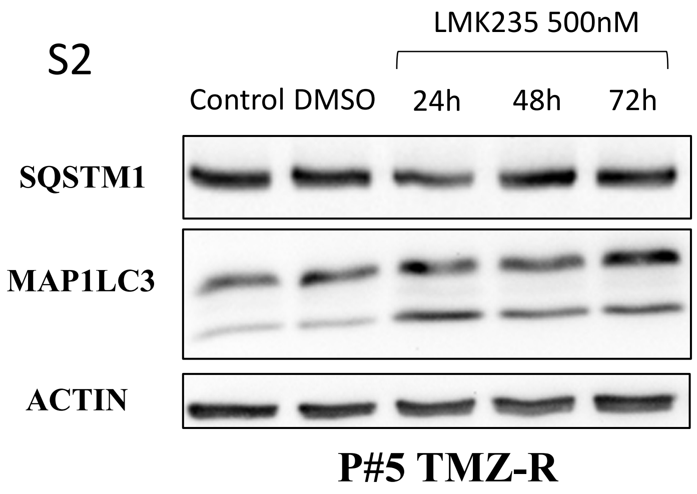

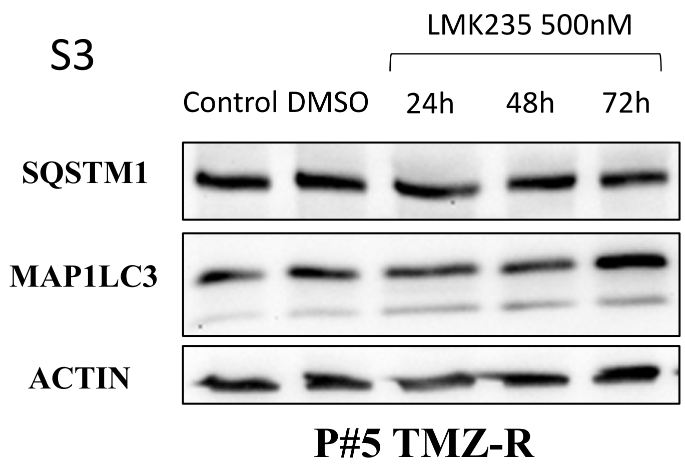

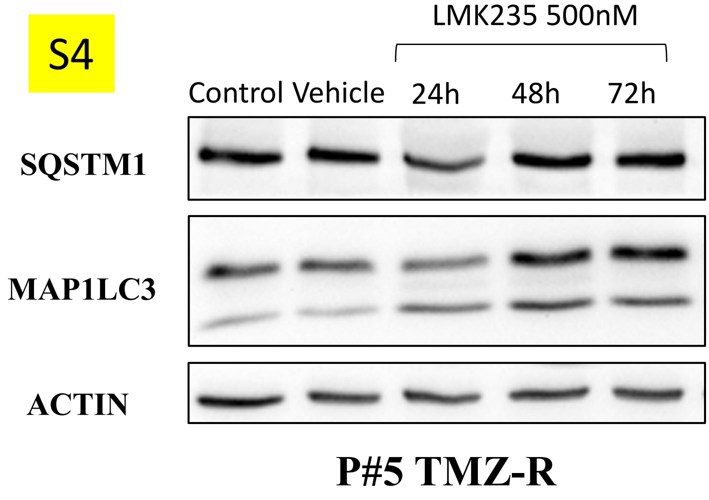

Whole image of WB in Fig. 5A

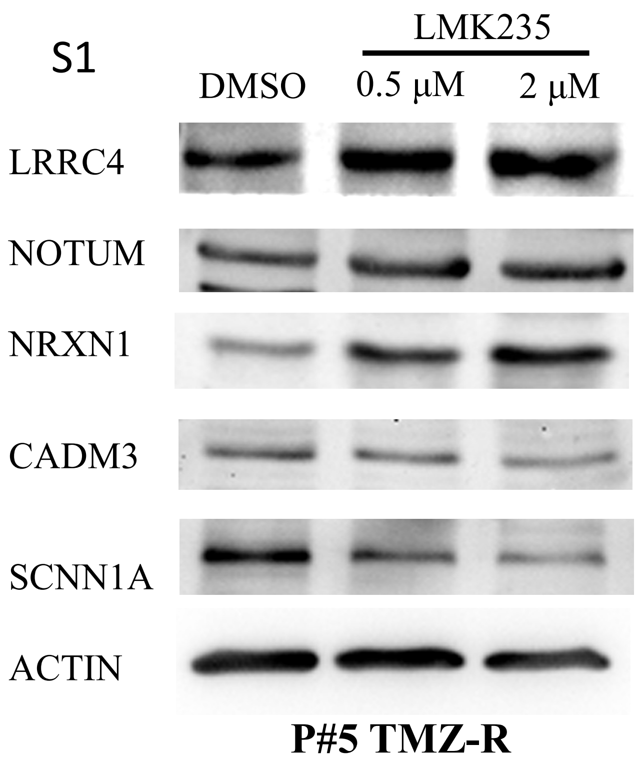

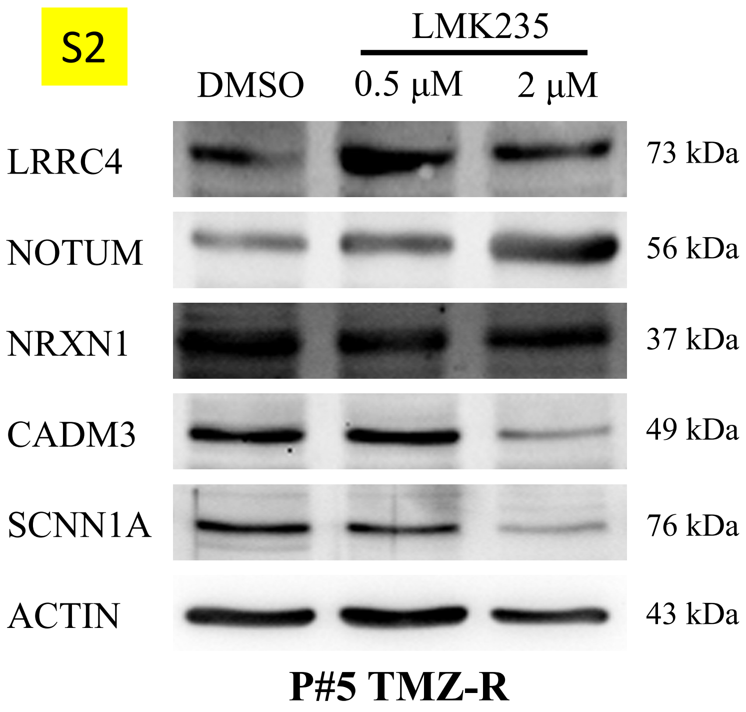

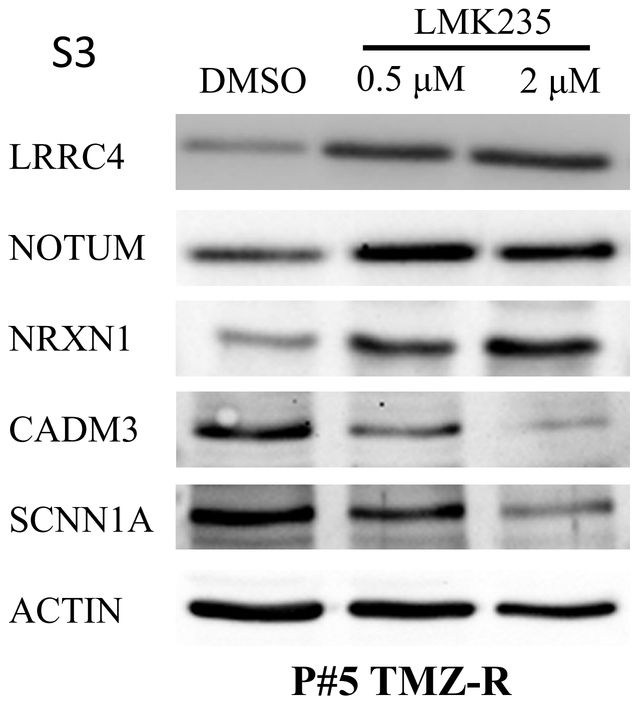

Whole image of WB in Fig. 5D

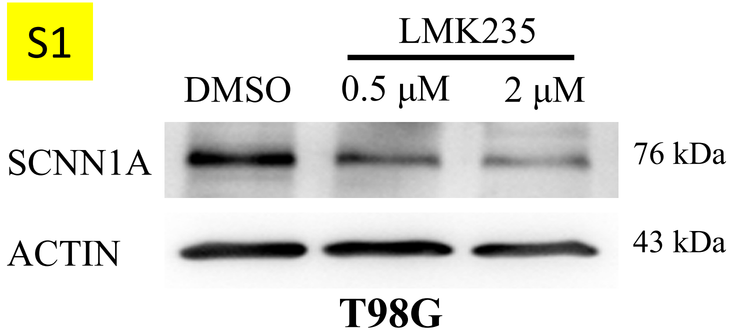

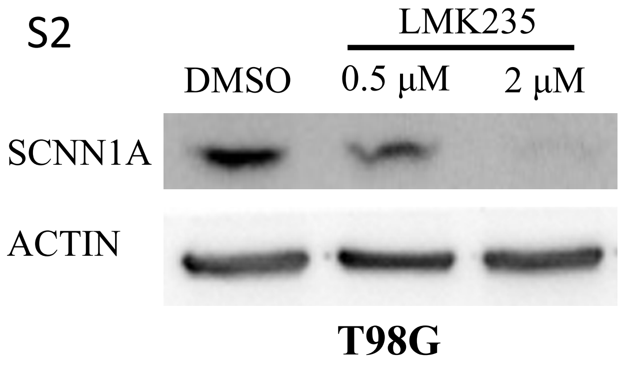

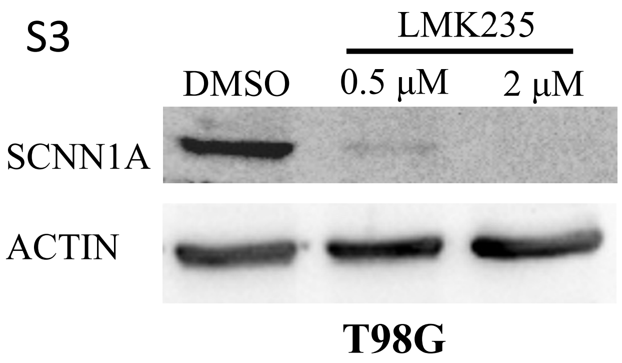

Whole image of WB in Fig. 5G

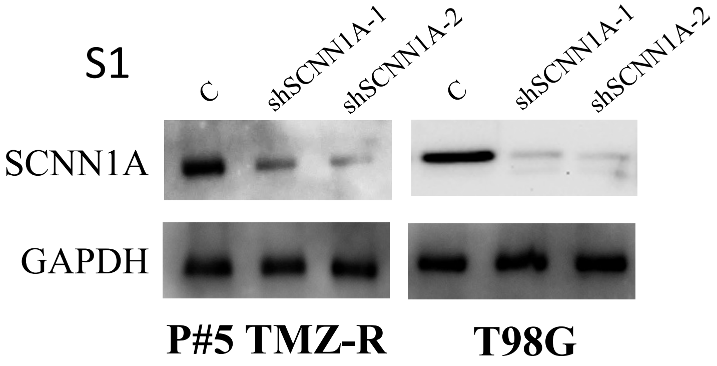

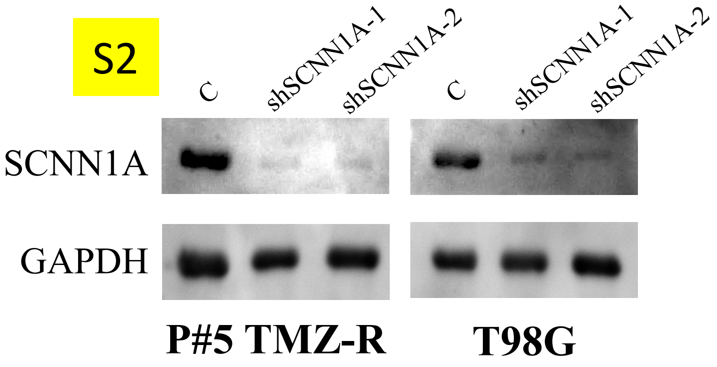

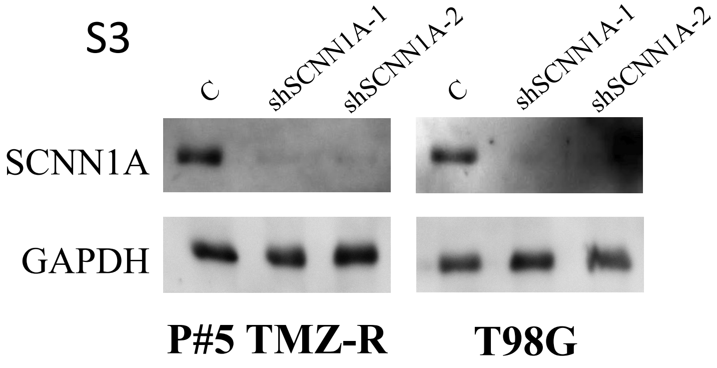

Supplement: Supplementary file 1 [file cancers-14-04537-s001.zip › Supplemental Materials File S1. Western blot.pdf]
