# Supplementary material for: A Selective Histone Deacetylase Inhibitor Induces Autophagy and Cell Death via SCNN1A Downregulation in Glioblastoma Cells
Source: Cancers (Basel). 2022 Sep 19;14(18):4537. doi: 10.3390/cancers14184537 (PMC9496778; doi:10.3390/cancers14184537)
Supplement: Supplementary file 1 [file cancers-14-04537-s001.zip › Supplementary Table S1.pdf]

cancers MDPI

Table S1. Primers used in qPCR analysis.

Gene Name		Sequence
NFASC	Forward	GGCAAAGCTGAAAACCAAGTCCG
	Reverse	TTCAGCCAGGAGACGGTGAGTT
IGSF11	Forward	CTACCTCCAACAGCTACTCAGG
	Reverse	CAGAAGACAGGTGCTGGTTCCA
NRXN1	Forward	GCTATCTTGGCAGGTCCTGTGA
	Reverse	ACATCCTCAGCCTCCGTATGCA
SELPLG	Forward	GAACCTGTCCACGGATTCAGCA
	Reverse	GTCAGTCGAGTTGTCTGTGCCT
CADM3	Forward	ATCCGGCACAAAGGAACCTAC
	Reverse	CGCCTTCTGCATTGATGATG
HLA-DMB	Forward	CACTTACACCTGTGTGGTAGAGC
	Reverse	GCAGACACAGAAACCTTCAGGG
HLA-DMA	Forward	GTTCTGCGAGTGGATGATCCAG
	Reverse	TGTTGGGCTTGCCAAACTCCAG
LRRC4	Forward	CGTGAAGTGGTTGCTGCCCAAT
	Reverse	GTTGCCTGCAACATTGGTCACC
NECTIN1	Forward	CTACCACATGGACCGCTTCAAG
	Reverse	CTTTGCAGGTGAGCTTCACGTC
CNTNAP1	Forward	GCCTTGTACTGCAACTGTGACG
	Reverse	CTCAGAAGTGGAGCGGTTCGTA
VSIR	Forward	AGATGCACCATCCAACTGTGTGG
	Reverse	AGGCAGAGGATTCCTACGATGC
CLDN3	Forward	GCCTTCATCGGCAGCAACATCA
	Reverse	AGCGAGTCGTACACCTTGCACT
STAT5A	Forward	GTTCAGTGTTGGCAGCAATGAGC
	Reverse	AGCACAGTAGCCGTGGCATTGT
IL27RA	Forward	GTGTGGGTATCAGGGAACCTCT
	Reverse	TCCTTCTGGACTCAGCTCACGA
PDGFB	Forward	GAGATGCTGAGTGACCACTCGA
	Reverse	GTCATGTTCAGGTCCAACTCGG
COL9A3	Forward	GAGTCCTCCCTGAAGGCGCTA
	Reverse	CTGCTCGCCTTTGTAGCCAGTG
MATN1	Forward	GGCTTCACTCTGAACAGCGACG
	Reverse	CCGTCAATGAGGAAGACCAGGT
SCNN1A	Forward	GTGCCTACATCTTCTATCCGCG
	Reverse	GTCTGAGGAGAAGTCAACCTGG
ANO1	Forward	GAAGCGGAAACAGATGCGACTC
	Reverse	CTGGCTTCGTATTCAGCTCTAGG
RTEL1	Forward	CCTATCCTGTCATGGAGAAGAGC
	Reverse	TCTCGGAGAAGCTGCCTTTGCT
GPER1	Forward	TTCCGCGAGAAGATGACCATCC
	Reverse	TAGTACCGCTCGTGCAGGTTGA
ADORA1	Forward	ATTGCTGTGGACCGCTACCTCC
	Reverse	CGCACTCAGATTGTTCCAGCCA
NOTUM	Forward	CTACTGGTGGAACGCAAACATGG
	Reverse	CGCACCACCTCCTGGATGATG

Gene Name		Sequence
FAM20C	Forward	CTGACTACTGCGAGGAGGTGAA
	Reverse	TCTCGTAGTGGTGACGGTCCAT
NTS	Forward	CAGCAGGGCTTTTCAACACTGG
	Reverse	CTCATACAGCTGCCGTTTCAGAA
GUCY2C	Forward	CAATCCAGAGACTACGACAGTGC
	Reverse	TGTGCCGTAGAACTTGGTCAGG
CD53	Forward	GCTATGCGAAAGCAAGACTGTGG
	Reverse	GTCAATCTGGCAGTTCAGGGTC
NCKAP1L	Forward	TCTGTGCTGAGCAGCGAAACCT
	Reverse	TGACTCTCAGCTCCTGGCTTGT
SCN1A	Forward	GGACTGTATGGAGGTTGCTGGT
	Reverse	GCAAGGTTGTCTGCACTAAATGAG
AGT	Forward	TGGACAGCACCCTGGCTTTCAA
	Reverse	ACACTGAGGTGCTGTTGTCCAC
TCEAL5	Forward	GACAGAATGCGAGGGAAAGCGA
	Reverse	TGTGGCTTGTCCTCACCTTCAG
BEX5	Forward	TGGTGAATACCAGGAGCCTGGA
	Reverse	CATCTCCTCCATGAACCGTTCC
CGA	Forward	TCCATTCCGCTCCTGATGTGCA
	Reverse	CGTCTTCTTGGACCTTAGTGGAG
ACTIN	Forward	GAGACCTTCAACACCCCAGC
	Reverse	ATGTCACGCACGATTTCCC
